# Histopathologic and Preneoplastic Changes in Tubal Ligation Materials

**DOI:** 10.3390/medicina59122117

**Published:** 2023-12-04

**Authors:** Ethem Ömeroğlu, Yaşar Ünlü, Ayşe Nur Uğur Kılınç, Tuğba Günler, Oğuzhan Günenc

**Affiliations:** 1 Clinic of Pathology, Konya City Hospital, University of Health Sciences Turkey, Konya 42020, Turkey; yasarunlu66@hotmail.com (Y.Ü.); aysenurugur@hotmail.com (A.N.U.K.); tubagurcan85@hotmail.com (T.G.); 2 Clinic of Obstetrics and Gynecology, Konya City Hospital, University of Health Sciences Turkey, Konya 42020, Turkey; oguzhangunenec@hotmail.com

**Keywords:** tubal ligation, epithelial hyperplasia, SCOUT, p53 signature

## Abstract

*Background and Objectives:* To investigate histopathological changes and serous carcinoma precursors such as secretory cell outgrowths (SCOUTs) and p53 signature in the bilateral tubal ligation (BTL) materials used during cesarean section (S/C). *Materials and Methods*: In total, 138 patients underwent S/C and tubal sterilization (TS) between October 2020 and May 2021 at Konya City Hospital. Patients’ data were obtained from the hospital’s system. All data and findings were investigated and statistically evaluated. *Results:* The mean age was 34.62 years (22–44), the mean gravity was 4.89 (2–15) and the mean parity was 3.46 (1–10). In total, 5.79% SCOUT, 7.24% atypia and 9.42% p53 signatures were observed. Significant correlations were shown between the epithelial cell lineage and age between Ki-67, SCOUT, and gravity; between the Ki-67 results and gravity and parity; and between the p53 score and age. *Conclusions*: TS is a common, safe, and effective method worldwide. Today, BTL is increasing along with increasing S/C ratios. In addition to the reduced risk of ovarian cancers with ligation alone, precursor lesions such as hyperplasia, SCOUT, p53 signature, and STIL/Serous tubal intraepithelial carcinoma (STIC) are encountered in the ampulla materials obtained. Considering the low rates of re-anastomosis, tubal excision may be recommended instead of ligation in women of relatively higher gravity and age.

## 1. Introduction

The uterine tubes are located bilaterally in the upper part of the uterine cavity and are, on average, 11–12 cm in length. Also known as the oviduct or Fallopian tube, these tubes consist of the infundibulum (fimbria), ampulla, isthmus, and intramural segments. They are embryologically derived from the coelomic epithelium via the paramesonephric ducts. In its normal histology, there are three types of epithelial cells: ciliated, secretory, and intercalate (peg). The appearance of these cells may change due to cyclic hormonal changes and the use of oral contraceptives [1].

Tubal sterilization (TS) is a highly effective, widely used, safe, and permanent birth control method. It is usually preferred by women who do not have fertility aspirations or who have completed them. This method is applied around 700,000 times a year in the USA [2]. The number and rate of TSs are increasing, with more than 190 million TSs administered globally in 2017 and more than 220 million administered in 2022 [2,3]. While the rate of preference for this method is 23.7% in the world, it reaches 4.3% in the USA [1,4]. In addition to that, TS has been found to decrease the risk of any ovarian cancer by 13% to 41% [5].

Routine pathologic examinations of TS materials reveal different, normal histopathologic margins and preneoplastic/neoplastic changes. Hyperplasia that develops in normal mucosal cells is in the form of the stratification and formation of small tufts and may show varying degrees of cytologic atypia [6,7]. Hyperplasia may be associated with conditions such as inflammation, excess estrogen, ectopic pregnancy, and neoplasia. It can be present in up to 83% of bilateral tubal ligation (BTL) and salpingectomies performed in benign and malignant conditions [6]. Most cases show only mild atypia and, in such settings, the presence of small papillary clusters and nuclear stratification should not be misinterpreted as a premalignant change. Mitotic figures are usually absent or few in number. Cells preserve cilia and the nuclear/cytoplasmic ratio [6].

The presence of at least 30 or more secretory cells is referred to as “secretory cell outgrowths (SCOUT)”. “p53 signatures” are abnormal p53 immunostaining in at least 12 consecutive secretory nuclei that appear normal. Serous tubal intraepithelial lesion (STILs) have been identified in the fallopian tube. STILs are lesions that fail to be classified as serous tubal intraepithelial carcinoma (STIC) due to limited proliferation (p53 positive-Ki-67 < 10% or p53 negative Ki-67 > 10%) [8,9]. STICs are associated with nuclear enlargement, a loss of polarity, increased nuclear atypia, abnormal p53 expression, and increased proliferative activity (Ki-67) [9,10]. 

Epithelial ovarian carcinoma is the second most common gynecologic malignancy in the United States and the most common cause of death in this group of tumors [11].

High-grade ovarian serous carcinoma (OSC) is responsible for approximately 70% of ovarian cancers. Most of them are bilateral. Low-grade OSC is uncommon (<5% of all ovarian cancers) [10,12]. High and low-grade OSCs are said to have different carcinogenesis origins [13]. In many studies, it has been reported that the risk of the development of ovarian cancer decreased by 42–77% with salpingectomy [14]. 

## 2. Materials and Methods

In our study, 138 patients who underwent BTL during S/C between October 2020 and May 2021 at Konya City Hospital were evaluated. For each patient, tubal ligation tissues were cut to 4–5 microns and prepared for hematoxylin–eosin and immunohistochemistry studies. An automated device (Leica Biosystems Melbourne Pty Ltd., Bond-max, M212536, 2014, Melbourne, Australia) was utilized for immunohistochemistry. The anti KI-67 antibody for immunohistochemistry (Mouse monoclonal, Biogenex, The Hague, The Netherlands), anti p53 (Mouse monoclonal, Zeta, Sierra Madre, CA, USA), and the Bond Polymer Refine Detection Kit (LeicaBiosystems, Newcastle Upon Tyne, UK) were used. The exhaustive protocol was attained from the anti-Ki-67 and anti-p53 product datasheets. Diaminobenzidine (DAB) and hematoxylin counterstaining were performed for visualization. The stained preparations were then sealed with a coverslip using entellan. 

Ethics committee approval of the study was obtained from Karatay University Faculty of Medicine, Pharmaceuticals and Medical Devices External Board on 24 January 2023, with the decision number 2023/036.

The cell layers observed most frequently and second frequently in the long-axis regions of the tubal epithelium were grouped separately as ≤2 and ≥3. Inflammation, intra-epithelial lymphocytes, papillary hyperplasia, tufting, atypia, SCOUT, p53 signature, and STIL conditions were evaluated as present/absent [8].

The number of cells presenting immunohistochemical staining with Ki-67 was evaluated by modifying the methods described in the literature. The scores were evaluated according to the number of cells with positive Ki-67 staining; <10% = score 1, 10–25% = score 2, >25% = score 3, cells staining with p53; 0 = f score 0, <10% = score 1, 10–25% = score 2, 25–50% = score 3, >50% = score 4 [15].

The descriptive statistics mean, standard deviation for quantitative variables, and frequency and percentage for categorical variables were given. For the analysis of categorical variables, Chi-square and Fisher tests were utilized, and for the analysis of numerical variables, a *T*-test and analysis of variance were utilized. Analyses were performed with the R 4.2.2 program and *p* < 0.05 was considered significant. To estimate the independent contribution of each variable to the maximum cell layer sequence, we applied a stepwise multiple logistic regression analysis model. All the variables that were significant at *p* value in the univariate analysis were considered for the model odds ratio and were calculated for each variable.

## 3. Results

In total, 138 women who underwent BTL during S/C were included in this study. The mean age was 34.62 (22–44), the mean gravity was 4.89 (2–15), and the mean parity was 3.46 (1–10). In the extended period, which includes the study period (between 15 August 2020 and 31 December 2021), there were 4568 S/Cs, 643 BTLs, and 2 re-anastomoses in our institution. 

**Figure 1:**
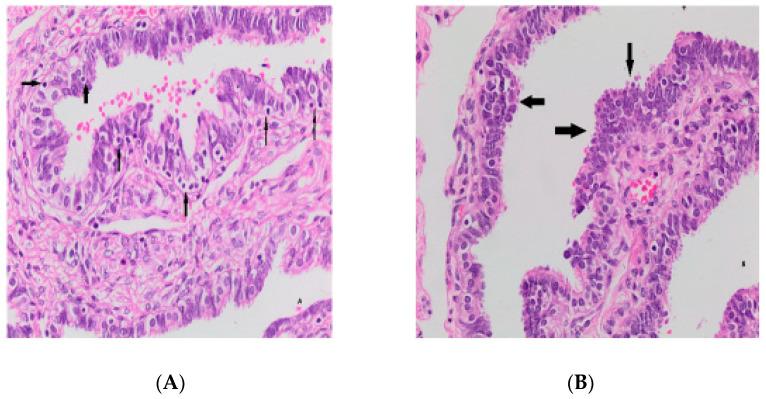
Intra-epithelial lymphocytes (arrows) (**A**); epithelial hyperplasia (arrows) (**B**).

Histologic findings related to the epithelial cell sequence, inflammation, and other epithelial changes are shown in the tables (Table 1 and Table 2, Figure 1). 

Immunohistochemically obtained Ki-67 and p53 scoring results, SCOUT, p53 signature, and STIL results are given in the tables (Table 2). In the evaluations made in terms of epithelial hyperplasia, there were significant correlations between cell sequence and age (more in the higher aged, *p* = 0.044) and cell sequence and the Ki-67 score (*p* = 0.044), while no correlation was found between cell line and gravity, the parity p53 score, p53 signature and SCOUT (*p* > 0.05).

**Figure 2:**
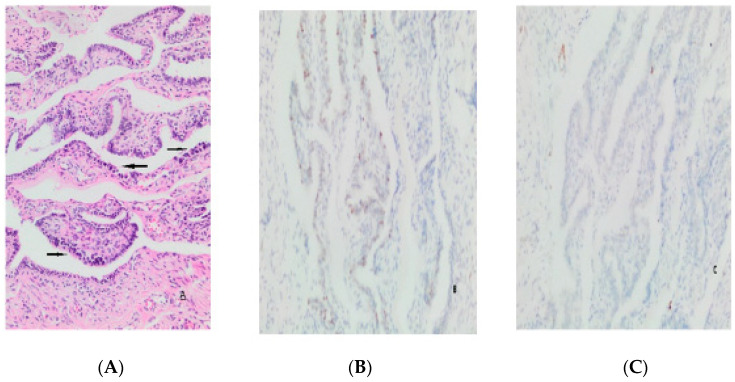
Atypical changes (arrows) (**A**), very low p53 (**B**), and KI-67 (**C**) values.

There was a significant (*p* = 0.011) association between the SCOUT findings and low gravity, whereas no association was found between age and parity (*p* > 0.05). Atypia was more common in those with higher cell sequences (*p* = 0.002). No correlation was found between p53, p53 signature, and SCOUT (*p* > 0.05) (Figure 2). The P53 score increases with increased age (*p* = 0.036). There was no correlation between gravity and parity and scores and p53 scoring (*p* > 0.005) (Table 3).

**Figure 3:**
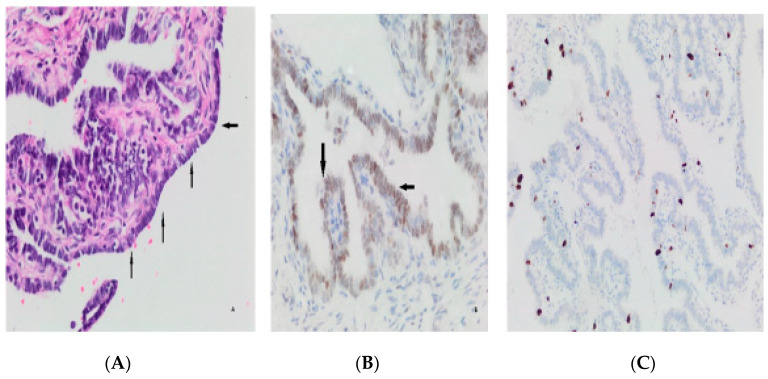
Secretory cell outgrowths (SCOUTs) (arrows) (**A**), p53 signature (arrows) (**B**), low Ki-67 index Score 1 (**C**).

**Figure 4:**
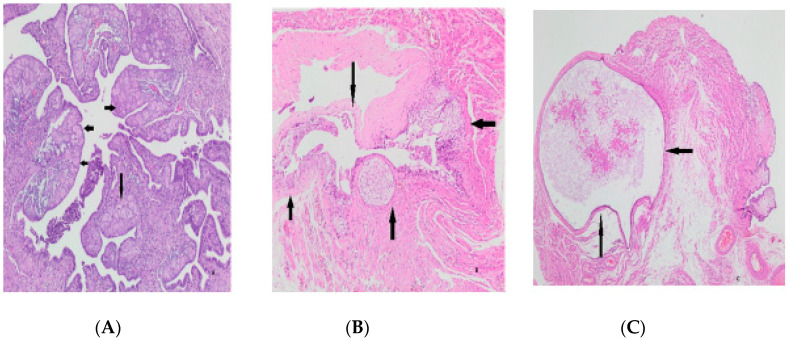
Extra findings. Decidua (arrows) in tubal tissue (**A**), Walthard remnants (arrows) (**B**), paratubal cyst (arrows) (**C**).

Higher Ki-67 scores were obtained in those with fewer gravities and parities, *p* = 0.016 and *p* = 0.049, respectively. There was no correlation with age (*p* > 0.05) (Figure 3). No correlation was found between Tufting, SCOUT, p53 signature, atypia, intraepithelial lymphocyte and infiltration findings, and age, gravity, and parity (*p* > 0.05) (Figure 3). Additional pathologic findings were a paratubal cyst in 10 cases (7.24%), decidua in 4 cases (2.89%), Walthard nest in 5 cases (3.62%), a paratubal cyst with Walthard nest in 2 cases (1.44%), and endometriosis and calcification in one case (0.72%) (Figure 4). Logistic regression for the maximum cell layer sequence (multivariable analysis) is shown in Table 4.

## 4. Discussion

In our study, we included 138 patients with BTL during S/C. Tubal excision is usually performed secondary to surgery, but it is also performed for primary sterilization. In a 7-year retrospective study published in 2001, TS was performed as an additional operation in 358 of 630 cases (56.82%) [16]. Tubal ligation is a frequently used method, especially during cesarean section. Regarding the cesarean section delivery rate, Turkey ranks first with 54.9%, followed by Korea at 45% and Poland at 38.9% [17]. In a 20-year study, tubal ligation during S/C increased from 5% to 24.7% [18]. In a study conducted in the United States, it was reported that bilateral salpingectomy was performed in 397,260 (10.4%) and BTL in 203,400 (5.3%) of 3,813,823 female patients who delivered via cesarean section in approximately three years [19]. While its efficacy and safety were recognized in most studies, side effects were reported at very low rates. In follow-up observations after cesarean section and tubal excisions performed in the postpartum period involving 435 cases, 26.2% of whom underwent tubal ligation, pregnancy developed at a rate of 0.7% [20]. Pregnancy was most commonly ectopic. In a study conducted in 2000, it was revealed that 700 thousand TSs were performed annually in the USA, that ectopic pregnancy occurred most often in unsuccessful applications, and that the risk of developing ovarian cancer decreased [8]. 

Moreover, tube ligation is suitable for patients who want to perform re-anastomosis for various reasons. In a study conducted with 80 patients, pregnancy occurred in 28 (47.4%) of the cases [2]. Pregnancy occurred in 8 (28.6%) of 28 patients who underwent re-anastomosis in 2021 [21]. 

The pathologic examination of tubal tissues was limited in the early years and was mostly focused on whether the correct/adequate material was obtained. Over time, histologic changes, especially the tubal lining of the ovaries, were examined in detail for preneoplastic changes due to their association with ovarian cancers. In 2010, 548 (79%) tubal ligation materials were sent to pathology in a clinic and 3 of them were found to contain insufficient tissue [20]. 

Tubal ligation is increasing gradually in our country and reached a rate of 10% in 2018 [22]. The rate of tube ligation during C/S is 14.07% in the institution we work in. The rate of re-anastomosis is quite low at 0.31%.

Comparative studies were conducted between the bilateral total or partial/ligation of the tubes under different parameters. Even though the follow-up period was as brief as one year, there were no differences in both the hormonal values (AMH, FSH, AFC, VI, FI, VFI) and OvAge and ovarian volumes of patients who underwent 50 bilateral total salpingectomies and 52 BTLs [23].

With the pathology data available, it has been demonstrated that up to 70% of ovarian cancers originate from the fallopian tubes. A study conducted in Sweden between 1973 and 2009 demonstrated that 251,465 women who underwent bilateral salpingo-oophorectomy for benign reasons during gynecological surgery had a lower risk of ovarian cancer than those who underwent BTL [24]. In the study, which lasted for about 14 years and had a considerably high number of cases (1,132,914 patients), TS was performed in 264,048 patients. As a result of this study, despite a 20% lower risk of developing high-grade OSC, there was no reduction in the risk of low-grade OSC [25].

Histopathological and preneoplastic transformations in the tubes were usually made in neoplastic patients compared to the control group, and in operative materials covering the fimbrial end. There is a limited number of studies on the tubal ampulla materials obtained as a result of TS, a widespread contraceptive method in the world, for the above purposes alone. 

Within the framework of normal histology, mast cells, neutrophil polymorphs, plasmacytes, and lymphocytic inflammatory cells can be observed in the tuba [26]. In a study with a large case series, Hunt et al. detected mast cells commonly in the material of 287 cases; however, the rate of lymphocytic inflammation was low. They reported that the rate of lymphoid follicles was 2.1% [27]. In our study, the rate of inflammatory cells was 33.34%, while the rate of intraepithelial lymphocytes was 14.492%. In the examination of the tubas of 201 patients, 60% of which had total abdominal hysterectomy and bilateral salpingo-ooferectomy (TAH-BSO), salpingitis was observed in 10.19%, hydrosalpinx in 7.86%, and pyosalpinx in 0.29% [28]. In a study conducted in contrast, salpingitis was observed to be as low as 1.9% in a follow-up of 72 patients after hysterectomy [10]. The salpingitis rate was found to be 7.24% in our study, as opposed to two studies with such different rates.

Tubal epithelial hyperplasia in nonneoplastic and neoplastic cases has long been of interest. In 1989, Susan et al. found epithelial hyperplasia in 68.7% of 99 cases with serous borderline [28]. While the rate of epithelial hyperplasia was 3% in a study of 200 non-neoplastic cases [4], the rate of cases with epithelial cell line >3 was 1.4% in another overlap [10]. In our study, the rate of cases with epithelial cell sequence ≥3 was 13.05%, and that of papillary hyperplasia was 5.07%. 

Again, in the study conducted by Hunt et al., atypia was detected in 7.3% of patients [27].

In a study by Fatemeh Sari Aslani, with 34 patients with ovarian neoplastic and 72 control patients, the rate of atypia in the control group was 0%. Significant differences were shown between the cases and the control group in terms of tubes, especially >3 cell layer thickness, atypia, mitosis, cribriform, and tufting (*p* < 0.05) [9]. The rate of atypia in our study was (7.24%). Atypia was more common in patients with higher cell sequences (*p* = 0.002). There was no correlation between the p53 scoring values, p53 signature, and SCOUT (*p* > 0.05).

In a study on Ki-67 scoring by Khun et al., 41 cases had STIC, 35 had HGSC (high-grade serous carcinoma), and 42 cases were in the control group; Ki-67 rates were significant between STIC and the control group, while no significant relationship was found between STIC and HGSC. There was no difference in the Ki-67 labeling rates for various regions of the tuba [29]. Significant correlations between the epithelial cell lineage and Ki-67 and between the Ki-67 results and gravity and parity were found in our study.

Studies on preneoplastic changes such as SCOUT, p53 signature, and STIL or preinvasive changes such as STIC have yielded different results in the ampulla and fimbria regions. In this study, the fimbriae are closer to the ovary than the ampulla and are more exposed to follicular fluid. The fimbriae were also shown to produce more CYYR1, SALL1, FOXP2, TAAR1, AKR1C2/C3/C4, NMBR, ME1, and GSTA2 genes, which are effective in antioxidant and inflammation pathways compared to the ampulla [30]. In a study conducted with 25 cases as the control group, 24 with OSC, and 75 with BRCA (+), the SCOUT rates were 12%, 83%, and 18%, respectively. SCOUT was more frequently observed at the fimbrial end than proximally. In parallel, the ratio of occurrence of SCOUT in the fimbriae and ampullae is 5:1 in cases with a higher p53 staining rate and 1.3:1 in cases with a lower p53 staining rate [30]. In a study of 34 cases of ovarian serous tumors with 72 in the control group, a significant difference in favor of tumor cases was observed in both bilateral fimbriae ((*p* = 0.012) and (*p* = 0.004)) and bilateral ampullae ((*p* = 0.012) and (*p* = 0.000)) [9]. In our study, the SCOUT rate was found to be 5.79%.

Precursors of HGSC containing the p53 signature and STIC occur most frequently in the fimbriae of the tuba. As mentioned above, this region is directly exposed to ovulation, WNT, and Notch signaling. Therefore, the fimbria is considered a high and the ampulla a low-risk epithelial region prone to transformation [31]. In a study, p53 mutation was detected using PCR and immunohistochemistry in 10 normal patients, 14 patients with carcinoma, and 1 patient with borderline, but only in 57% of patients with cancer. PCR was used to confirm seven of these [32]. The rate of patients with a significant p53 score was 3.62% in our study. The score increases with an increase in age (*p* = 0.036). We noted no correlation between gravity and parity counts and the p53 scoring in our study (*p* > 0.005). In a prospective study of 67 gynecologic and 46 obstetric patients, Tsutomu Ida et al. showed a lower incidence of p53 signatures in women who had given birth, were young, premenopausal, and had a history of pregnancy. p53 signatures were 21 (88%) in fimbriae and 3 (12%) in ampullae [12]. In a study of 32 cases with OSC, with 31 in the control group, p53 signatures and STIC were most frequently seen in serous carcinomas. p53 signatures and STIC were always seen at the fimbrial end [33]. In our study, the p53 signature rate was 9.42% and no correlation was found between age, gravity, and parity (*p* > 0.05). In 495 cases, 110 of which were malignant sectioning and whose fimbriated end was extensively examined (SEE-FIM) STIC was diagnosed in 13 cases, including 12 in fimbriae. STIC was not observed in benign gynecologic cases [9]. STIC was diagnosed in 2.4% of 85 elective hysterectomy patients over 40 years of age in Mexico after the examination of their tubes [34]. In the salpingectomy materials of 400 patients carrying the BRCA1/2 mutation, 1.5% had invasive serous carcinomas, 3.5% had STICs, and 1.3% had STILs [35].

In Khun’s study, 11 (26.8%) of the STIC findings were composed of a flat and highly atypical single-layered epithelium, 10 (24.4%) were composed of a multi-layered epithelium, and 20 (48.8%) were tufted [29]. STIC was not encountered in our study, whereas STIL was found in only one case (0.72%).

Meta-analyses of observational epidemiologic studies have repeatedly emphasized that tubal ligation is associated with an overall reduced risk of ovarian cancer [12]. Even if it is only the ligation of the ampulla, it is the right approach to consider this advantage.

Bhattacharya et al. examined the tuba of 201 patients with 60% TAH-BSO and found that, while only one case was neoplastic, paratubal cysts, ectopic tubal gestation, and Walthard Cell Nests were the most common [28]. In a series of 287 cases, Hunt et al. reported stromal and structural changes such as fibrosis in 35.5%, intramuscular edema in 12.5%, inclusion cysts in 7.7%, Walthard cell nests in 5.2%, tibial pigments in 5.1%, the Wolffian duct remnant in 4.5%, decidualized stroma in 3%, decidualized stroma and endosalpingiosis in 2.4%, and metastatic carcinoma infiltration in 1.4% [27].

In the study of Fatemeh Sari Aslani, 2.8% endometriosis, 1.9% salpingitis, 0.9% hemangioma, and 0.9% calcification were found in 106 tubas in the control and study groups [10]. In various studies, endometriosis rates have been reported to be between 1.4% and 43.3% [36]. In our study, aside from inflammation and epithelial changes, additional findings such as decidua, Walthard’s nest, and calcification were detected in the same types and at similar rates on average. Further cross-sectioning and, if necessary, the sampling of all material when examining tubal sterilization materials will make it easier to demonstrate the changes mentioned above. 

## 5. Conclusions

TS with the possibility of re-anastomosis is a widely used, safe, and effective method. Tubal ligation is also increasing with the increasing S/C rates in the world. Numerous comparative studies (malignant versus benign groups) have been conducted, especially based on the relationship between HGSC development and the tuba. In these studies, findings related to the fimbria and ampulla of the tuba were also evaluated. In addition to the reduced risk of ovarian cancers with ligation alone, a wide range of pathologic findings such as hyperplasia, SCOUT, p53 signature, and STIL/STIC are encountered in the ampulla materials collected. In our study, significant correlations were shown between age and the epithelial cell lineage and p53; between Ki-67 and the epithelial cell lineage, gravity and parity; and between SCOUT and gravity. Considering the low rates of re-anastomosis, tubal excision may be recommended instead of ligation in women of relatively higher gravity and age. With the evaluation of the above-mentioned findings, valuable data can be obtained both in the control and follow-up of patients and in prospective scientific studies.

## Figures and Tables

**Table 1:** Descriptive data related to epithelial cell sequence numbers and *p*-values.

		NCL ≤ 2	NCL ≥ 3	*p*-Value
Age	<25 years	2 (1.45%)	1 (0.72%)	0.024
	26–30 years	19 (13.77%)	7 (5.07%)	
	31–35 years	44 (31.89%)	4 (2.89%)	
	>40 years	16 (11.59%)	1 (0.72%)	
Gravity Number	≤3	18 (13.04%)	5 (3.63%)	0.90
	4–6	83 (60.14%)	11 (7.98%)	
	6–9	16 (11.60%)	1 (0.72%)	
	≥10	3 (2.17%)	1 (0.72%)	
Parity Number	≤3	86 (62.32%)	2 (1.44%)	0.71
	4–6	28 (20.29%)	3 (2.18%)	
	6–9	5 (3.63%)	1 (0.72%)	
	≥10	1 (0.72%)	1 (0.72%)	
Papillary Hyperplasia	Absent (n)	115 (83.34%)	5 (3.63%)	0.92
	Present (n)	16 (11.59%)	2 (1.44%)	
Tufting	Absent (n)	104 (88.41%)	16 (11.59%)	0.003
	Present (n)	10 (7.2%)	8 (92.8%)	
Inflammatory cells	Absent (n)	84 (60.86)	8 (92.8%)	0.029
	Present (n)	36 (39.14%)	10 (7.2%)	

(NCL: Number of cell layer sequence).

**Table 2:** Ki-67, p53 scoring, SCOUT, p53 signature and STIL counts.

Parameters	Scores	N/%
Ki-67	1	127 (92.02%)
	2	8 (5.79%)
	3	3 (2.17%)
p53	0	50 (36.23%)
	1	38 (27.53%)
	2	27(19.56%)
	3	18 (13.04%)
	4	5 (3.62%)
SCOUT	0	130 (94.21%)
	1	8 (5.79%)
STIL	0	137 (99.28%)
	1	1 (0.72%)
p53 signature	0	125 (90.58%)
	1	13 (9.42%)

SCOUT (secretory cell outgrowths); STIL (serous tubal intraepithelial lesion).

**Table 3:** *T*-test results (*p*-values) of variables.

	SCOUT	p53 Score	p53 Signature	Kİ-67 Score
Age	0.4	0.036	0.2	0.2
Gravity	0.2	0.8	0.7	0.016
Parity	0.6	0.7	>0.9	0.049
Atypia	0.5	0.2	0.6	0.3
NCL	0.3	0.7	>0.9	0.7
Papillary Hyperplasia	<0.9	0.6	0.5	>0.9
Tufting	0.14	0.030	>0.9	0.6
Inflammatory cells	0.4	0.3	<0.9	0.7
SCOUT	-	0.3	0.028	>0.9
p53	0.3	-	<0.001	0.053
p53 Signature	0.028	<0.001	-	0.020
Ki-67	>0.9	0.053	0.02	-

IEL (Intraepithelial lymphocyte), SCOUT (secretory cell outgrowths), STIL (serous tubal intraepithelial lesion).

**Table 4:** Logistic regression for number of cell layer sequence (multivariable analysis).

Variant	N	OR ^1^	95% CI ^1^	*p*
Age	138	0.88	0.79, 0.98	0.024
Gravity	138	0.98	0.74, 1.23	0.90
Parity	138	0.94	0.63, 1.27	0.71
p53	138			0.17
0		—	—	
1		0.30	0.06, 1.07	
2		0.28	0.04, 1.17	
3		0.44	0.06, 1.90	
4		0.00		
Ki-67	138			0.65
0		—	—	
1		0.92	0.05, 5.67	
2		0.00		
Tufting	138			0.003
0		—	—	
1		5.20	1.76, 15.3	
p53 signature	138			0.52
0		—	—	
1		0.53	0.03, 2.96	
SCOUT	138			0.35
0		—	—	
1		2.37	0.33, 11.4	
Papillary Hyperplasia	138			0.92
0		—	—	
1		1.12	0.06, 7.12	
Atypia	138			0.088
0		—	—	
1		0.00		
Inflammation	138			0.29
0		—	—	
1		0.58	0.21, 1.63	

^1^ OR = Odds Ratio, CI = Confidence Interval. SCOUT (secretory cell outgrowths), STIL (serous tubal intraepithelial lesion).

## Data Availability

Data are contained within the article.
